# Correlation of Red Blood Cell Morphology with Serum Cobalamin and Folate Concentration in Dogs with Chronic Diarrhea: A Retrospective Study

**DOI:** 10.3390/metabo14120657

**Published:** 2024-11-25

**Authors:** Argyrios Ginoudis, Anna Maria Ioannidou, Dimitra Pardali, Asteria Tsikna, Zoe Polizopoulou

**Affiliations:** Diagnostic Laboratory, School of Veterinary Medicine, Faculty of Health Sciences, Aristotle University of Thessaloniki, 54643 Thessaloniki, Greece; agkinou@vet.auth.gr (A.G.); ioannidouannie@gmail.com (A.M.I.); dpardali@vet.auth.gr (D.P.); asteria_vet@yahoo.com (A.T.)

**Keywords:** anemia, blood smear, canine, complete blood count, hematology, gastroenterology

## Abstract

**Background/Objectives**: Chronic diarrhea in dogs is a prevalent condition that significantly impacts canine health, often leading to weight loss, dehydration, and malnutrition. Diagnosing and treating chronic diarrhea is challenging due to its multifactorial nature, necessitating collaboration among veterinarians across various specialties. Measuring cobalamin and folate levels is a crucial diagnostic step for all dogs with chronic diarrhea. The role of these vitamins in erythropoiesis is well-documented in human medicine, where deficiencies are linked to erythropoietic disorders and megaloblastic anemia. This study explores the relationship between cobalamin and folate concentrations with hematologic parameters in dogs with chronic diarrhea to develop novel diagnostic methods that facilitate timely decision making. **Methods**: Forty-seven adult dogs with a history of chronic diarrhea (2019–2023) were included in the study. Upon presentation, complete blood count and measurement of cobalamin and folate concentrations were performed. The correlation of cobalamin and folate levels with erythrocytic parameters, including hematocrit (HCT), hemoglobin concentration (HGB), mean corpuscular volume (MCV), mean corpuscular hemoglobin (MCH), mean corpuscular hemoglobin concentration (MCHC), red cell distribution width (RDW), and reticulocyte count, as well as morphological changes in the blood smear were examined. **Results**: Serum cobalamin was significantly correlated with RBC (*p* = 0.032), HGB (*p* = 0.006), HCT (*p* = 0.005), and MCV (*p* = 0.022). Anisocytosis was significantly correlated with hypocobalaminemia (*p* = 0.002), while acanthocytosis correlated with normal cobalamin levels (*p* = 0.046). No correlation was found between serum folate and erythrocytic parameters or morphological changes. **Conclusions**: These findings emphasize cobalamin’s potential role in canine erythropoiesis, highlighting the need for routine evaluation and supplementation when necessary. Conversely, the lack of association with folate suggests it plays a less significant role in this species. These results underscore the importance of complete blood count in the diagnostic investigation of dogs with chronic diarrhea.

## 1. Introduction

Chronic diarrhea in dogs is a common and often debilitating condition characterized by repeated and persistent episodes lasting more than three weeks [1]. This condition can significantly affect the health and welfare of dogs, leading to weight loss, dehydration, and malnutrition [2]. Despite its frequent occurrence, chronic diarrhea in dogs presents a complex diagnostic and therapeutic challenge due to its multifactorial etiology, requiring extensive collaboration of the clinician with clinical pathologists, anatomic pathologists and veterinarians of other specialties in its investigation [3]. The diagnosis of chronic diarrhea in dogs requires a systematic and comprehensive approach, which often involves multiple diagnostic methods.

Laboratory tests, including a complete blood count (CBC), serum biochemistry, and urinalysis, are fundamental in the diagnostic process of categorizing enteropathy and the investigation of possible underlying diseases. Measurement of bile acid concentrations pre- and postprandially helps to confirm or exclude liver disease [4]. Canine-specific pancreatic lipase activity is a sensitive, specific, non-invasive method in dogs with suspected pancreatitis [5]. Fecal analysis is crucial for the detection of parasitic infections, bacterial overgrowth, and markers of inflammation such as calprotectin [6,7,8]. Further diagnostic techniques such as abdominal ultrasound, endoscopy, and biopsy are also required to assess the structural and functional integrity of the gastrointestinal tract [9,10]. Molecular diagnostic techniques, including polymerase chain reaction (PCR) and next-generation sequencing (NGS), offer accurate identification of infectious agents and genetic predispositions [11].

Specifically, serum concentrations of cobalamin and folic acid can help to identify the affected part of the small intestine and the possibility of exocrine pancreatic insufficiency (EPI) or bacterial overgrowth [12]. In dogs, chronic and severe small intestine disease and EPI are the predominant causes of cobalamin (vitamin B12) deficiency. In addition, hereditary cobalamin deficiency has been reported in several breeds of dogs [13]. Studies show that a significant proportion of dogs (82%) with EPI have cobalamin deficiency, a feature that is less evident in humans with EPI, likely due to species-specific variations in cobalamin absorption mechanisms. While humans rely primarily on the gastric mucosa for the supply of the intrinsic factor, dogs depend primarily on pancreatic cells [14].

Dogs with severe, chronic small intestine disease affecting the ileum may also have cobalamin deficiency. Like humans, dogs have substantial cobalamin stores, delaying the onset of deficiency despite inadequate absorption. Studies have shown that exogenously administered cobalamin has a longer half-life in healthy cats compared to those with gastrointestinal disease, highlighting the ability to replenish the body’s reserves [15]. Inherited cobalamin deficiency has been documented in specific dog breeds, such as the Giant Schnauzer, Beagle, Border Collie, Australian Shepherd, Yorkshire Terrier, and Chinese Shar-Pei [15,16,17,18,19,20]. The diagnosis of cobalamin deficiency is difficult and is based mainly on serum cobalamin concentration, despite limitations related to its unsatisfactory correlation with cellular cobalamin sufficiency. Elevated serum or urinary methylmalonic acid (MMA) levels, although indicative of cobalamin deficiency due to the role of cobalamin in the conversion of MMA into succinyl-CoA, are usually not measured, due to technical complexity and cost [21]. Thus, serum cobalamin concentration remains the main essential diagnostic tool in veterinary practice for the evaluation of chronic gastrointestinal symptoms or suspected cobalamin deficiency.

Folic acid, a water-soluble vitamin belonging to the B vitamin group (vitamin B9), is mainly found as folic acid polyglutamate, which is not absorbable. In the proximal small intestine, polyglutamate folic acid undergoes deconjugation by folate deconjugase, a jejunal brush border enzyme, resulting in folic acid monoglutamate being absorbed via specific folic acid carriers, also found in the proximal small intestine [22]. Severe disease processes may affect either polyglutamic folic acid deconjugation or folic acid monoglutamate absorption, leading to folic acid malabsorption. Persistent malabsorption of folic acid depletes folic acid stores, resulting in reduced serum folic acid concentrations. Similar mechanisms apply in cases of diffuse small intestine disease involving the proximal small intestine. Folic acid is synthesized by many species of bacteria and the increased presence of these bacteria (i.e., small intestinal dysbiosis) is thought to significantly increase serum folic acid concentrations [23].

The role of cobalamin and folate in erythropoiesis in humans has been well-established. The differentiation of erythroblasts requires sufficient amounts of both vitamins [24]. The mechanism of cobalamin- and folate-deficient anemia has been attributed to impaired DNA metabolism that results in disturbed maturation of the erythroid lineage in the bone marrow [25]. In dogs, there is no evidence that cobalamin or folate deficiency is connected to equivalent changes in the erythrocytic lineage, although one report that included dogs with inherited cobalamin malabsorption revealed anemia with poikilocytosis and anisocytosis and megaloblastic changes in the bone marrow [15].

Therefore, it has become clear that measurement of folic acid and cobalamin levels in dogs with diarrhea is essential. This study aims to investigate the correlation of red blood cell morphology with serum cobalamin and folate concentrations in dogs with chronic diarrhea, due to the significance of those vitamins both in the diagnostic investigation of chronic diarrhea and in erythropoiesis.

## 2. Materials and Methods

### 2.1. Study Population

Cases were retrospectively identified and retrieved from the archive of the Diagnostic Laboratory and the Companion Animal Clinic of the School of Veterinary Medicine, Aristotle University of Thessaloniki for the period of 2019–2023. Dogs that presented with a history of diarrhea lasting for more than 3 weeks and that underwent CBC and cobalamin and folate concentration measurement on presentation were included in the study. Dogs in which lymphoma or hypoadrenocorticism was identified as the cause of diarrhea as well as dogs with a history of recent cobalamin or folate supplementation were excluded from the study [26,27]. The epidemiological data are presented in Table 1.

### 2.2. Complete Blood Count

A CBC using EDTA anticoagulated whole blood as per clinic standard procedure was performed for every animal upon presentation using the automated analyzer ADVIA 120 Hematology and Cytometry System (Bayer HealthCare LLC., Diagnostics Division, Tarrytown, NY, USA). The clinic’s standard procedure involves drawing blood from the jugular vein and collecting it in 1 mL EDTA tubes. The erythrocytic parameters used in this study were red blood cell count (RBC, RI: 5.5–8.5 × 10^6^/μL), mean corpuscular volume (MCV, RI: 60.6–77.0 fL), hematocrit (HCT, RI: 37.1–55.0%), hemoglobin concentration (HGB, RI: 12.0–18.0 g/dL), mean corpuscular hemoglobin (MCH, RI: 19.5–24.5 pg), mean corpuscular hemoglobin concentration (MCHC, RI: 31.0–36.2 g/dL), red cell distribution width (coefficient of variation %) (RDW, RI: 11.9–14.5%), and reticulocyte count (RETIC, RI: <90.000/μL).

### 2.3. Blood Smear Examination

A Giemsa-stained blood smear from each dog was retrieved from the archive of the Diagnostic Laboratory and reexamined by two experienced observers using a Leica microscope DM1000 (Leica Microsystems, Wetzlar, Germany). According to standard procedures, every smear is prepared with the Wedge method, fixed with methanol and stained with a modified Giemsa stain. The morphological changes of the erythrocytes were observed at the monolayer of the smear with the immersion oil lens (×1000 magnification) and noted in a qualitative manner. Observed changes included anisocytosis (based on the assessment described by Gulati [28]), acanthocytosis, polychromasia, and echinocytosis.

### 2.4. Cobalamin and Folate Concentration

The concentrations of cobalamin (RI: 173–599 pmol/L) and folate (RI: 21.1–54.0 nmol/L) in serum samples were measured using the Chemiluminescent Immunoassay (CLIA) method (VET MED LABOR GMBH-Division of IDEXX Laboratories, Leipzig, Germany). The CLIA method employs specific antibodies that bind to vitamin B12 and folic acid, forming an antigen-antibody complex. This complex is then exposed to a chemiluminescent substrate, resulting in the emission of light proportional to the concentration of the target analytes. The emitted light is measured using a luminometer, and the intensity of the luminescence is directly related to the levels of cobalamin and folate in the samples.

### 2.5. Statistical Analysis

For the statistical analyses, a statistical software package (IBM Corp., released 2021: IBM Statistical Package for Social Sciences—SPSS for Windows, Version 28.0, Armonk, NY, USA) was used to calculate the Pearson’s and Spearman’s correlation coefficients of cobalamin and folate with the erythrocytic parameters. In order to assess the possible predictive value of morphological changes for low serum cobalamin and folate concentrations, the dogs were divided into groups. Two groups were based on cobalamin concentrations (Group A: Cobalamin concentration < 173 pmol/L, Group B: Cobalamin concentration ≥ 173 pmol/L) and a chi-square test was performed to examine the correlation of any observed morphological changes with hypocobalaminemia. A chi-square test was also performed in two different groups based upon folate concentration (Group C: Folate concentration < 21.1 nmol/L, Group D: Folate concentration ≥ 21.1 nmol/L) to examine the correlation of morphological changes with hypofolatemia.

## 3. Results

Forty-seven dogs (25 female and 22 male; median age: 5 years old) were included in the study.

Table 2 shows the Spearman’s correlation coefficient (ρ) of cobalamin with the erythrocytic parameters of the CBC.

**Figure 1:**
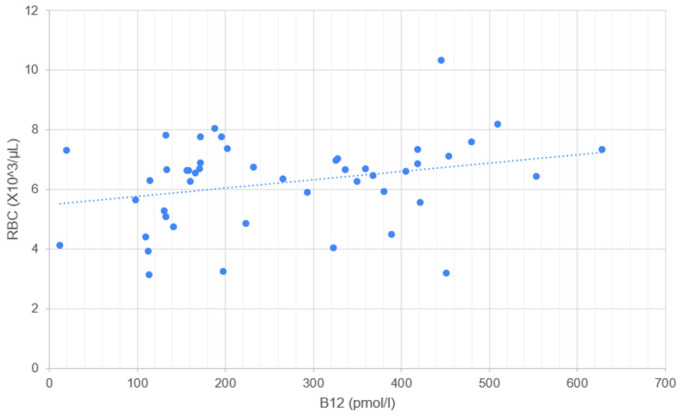
Scattergram illustrating the relationship between serum cobalamin concentration (B12, pmol/L) and red blood cell count (RBC, ×10^3^/µL). Each point represents an individual measurement, where the x-axis corresponds to the B12 concentration and the y-axis corresponds to the RBC concentration. The dotted line represents the trend line (linear regression) indicating the correlation between B12 and RBC.

**Figure 2:**
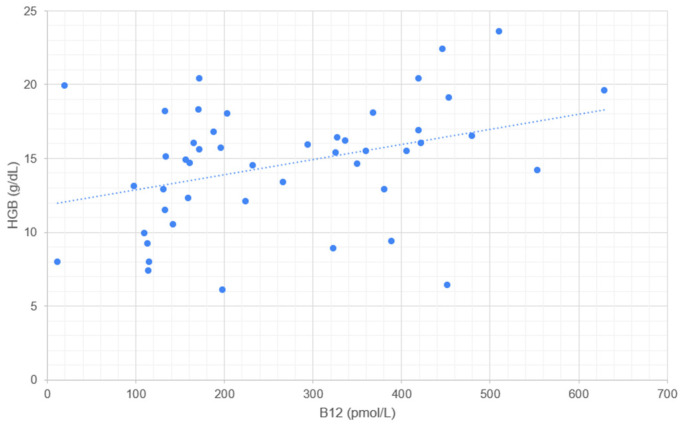
Scattergram illustrating the relationship between serum cobalamin concentration (B12, pmol/L) and hemoglobin concentration (HGB, g/dL). Each point represents an individual measurement, where the x-axis corresponds to the B12 concentration and the y-axis corresponds to the HGB concentration. The dotted line represents the trend line (linear regression) indicating the correlation between B12 and HGB.

**Figure 3:**
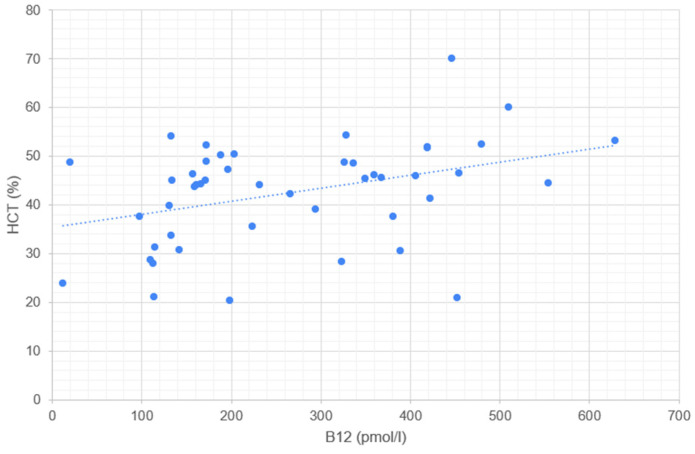
Scattergram illustrating the relationship between serum cobalamin concentration (B12, pmol/L) and hematocrit (HCT, %). Each point represents an individual measurement, where the x-axis corresponds to the B12 concentration and the y-axis corresponds to the HCT. The dotted line represents the trend line (linear regression) indicating the correlation between B12 and HCT.

**Figure 4:**
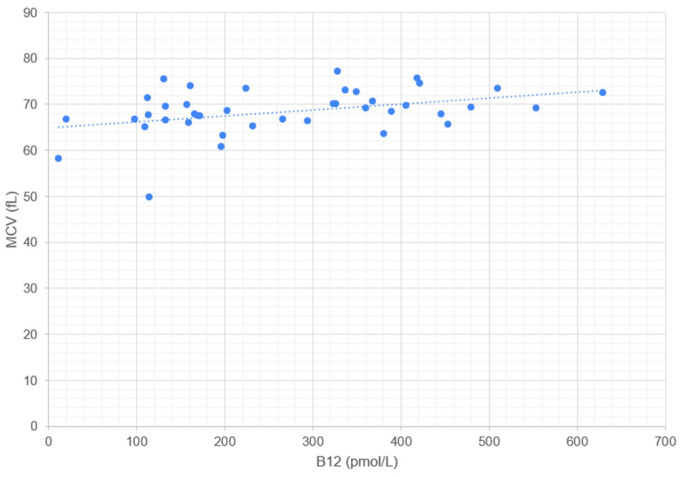
Scattergram illustrating the relationship between serum cobalamin concentration (B12, pmol/L) and mean corpuscular volume (MCV, fL). Each point represents an individual measurement, where the x-axis corresponds to the B12 concentration and the y-axis corresponds to the MCV. The dotted line represents the trend line (linear regression) indicating the correlation between B12 and MCV.

Serum cobalamin concentration correlates significantly with RBC (Figure 1), HGB (Figure 2), HCT (Figure 3), and MCV (Figure 4). No correlation was found between serum cobalamin and MCH, MCHC, RDW, and RETIC.

Table 3 shows the Spearman’s correlation coefficient (rho) of folate with the erythrocytic parameters of the CBC. Serum folate shows no correlation with the erythrocytic parameters of the CBC.

The patients were divided into two groups based on cobalamin concentration (Group A: Cobalamin concentration < 173 pmol/L, 19 dogs in total, Group B: Cobalamin concentration ≥ 173 pmol/L, 28 dogs in total) and a chi-square test was performed to examine the correlation of any observed morphological changes with hypocobalaminemia. A chi-square test was also performed in two different groups based upon folate concentration (Group C: Folate concentration < 21.1 nmol/L, 21 dogs in total, Group D: Folate concentration ≥ 21.1 nmol/L, 26 dogs in total). The morphological changes reported in the blood smear are summarized in Table 4.

Eight hypocobalaminemic dogs (42.1%) and one normocobalaminemic dog (3.6%) presented anisocytosis in the blood smear. Anisocytosis is significantly correlated with hypocobalaminemia (*p* = 0.002). Acanthocytosis was reported in 2 hypocobalaminemic dogs (10.5%) and in 11 normocobalaminemic dogs (39.3%). Acanthocytosis is significantly correlated with normal serum cobalamin levels (*p* = 0.046). No other morphological change presented significant correlation with cobalamin levels. Group C and D did not show any significant difference in any morphological alterations. Table 5 shows the statistically significant correlations.

## 4. Discussion

In this study, a retrospective investigation of the correlation between cobalamin and folic acid concentrations with erythrocyte parameters and morphological characteristics in dogs suffering from chronic diarrhea was performed. Chronic diarrhea is known to potentially be associated with malabsorption of essential nutrients, leading to significant changes, including effects on the hematopoietic system. This study provides valuable insights into the specific roles and implications of cobalamin and folic acid deficiencies in the context of chronic diarrhea, highlighting key associations, clinical implications, and areas for future research.

A statistically significant positive correlation was found between cobalamin concentration and RBC and HCT, suggesting that cobalamin deficiency may lead to reduced erythrocyte count, as known in human nutritional anemias. In a study investigating the correlation of serum cobalamin concentration with anemia, no statistically significant differences were observed between dogs with normal cobalamin concentration and those with hypocobalaminemia in terms of the presence of anemia [29]. However, the population studied did not include dogs with chronic diarrhea, but all dogs with measured concentrations of the two vitamins, and the linear correlation between cobalamin and erythrocyte parameters was not investigated.

A significant positive correlation was observed between cobalamin concentration and HGB, which is considered to be a consequence of the correlation with erythrocyte count. This hypothesis is supported by the absence of correlation of MCH and MCHC with cobalamin. A linear correlation of serum cobalamin with HGB, has also been detected in humans [30]. This study, however, did not include any deficient individuals.

In contrast to the human literature, where cobalamin deficiency is accompanied by macrocytosis, a significant correlation was found between cobalamin levels and MCV (*p* = 0.022). This means that in the dog, lower cobalamin concentration is accompanied by a decrease in red blood cell volume (microcytosis). Microcytosis in dogs has been associated with iron deficiency anemias, anemia of chronic disease, protoporphyria, and liver disease [31,32,33].

For dogs diagnosed with cobalamin deficiency, supplementation is a critical intervention [34]. Parenteral administration of cobalamin was considered preferable in cases with significant gastrointestinal malabsorption to ensure adequate concentrations are achieved in the body; however, recent studies have highlighted the similar therapeutic value of oral administration [35]. Regular monitoring of cobalamin concentrations and hematological parameters is essential to guide the frequency and duration of supplementation, ensuring effective management of deficiency and associated hematological abnormalities.

There was a significant correlation between anisocytosis and hypocobalaminemia, highlighting the impact of cobalamin deficiency on erythrocyte size variation. Surprisingly, acanthocytosis was more prevalent in dogs with normal cobalamin levels (39.3%) compared to those with hypocobalaminemia (10.5%), indicating a significant correlation with normal cobalamin concentration. Acanthocytosis is attributed to alterations in the erythrocyte membrane [36]. Based on this fact, our results indicate that cobalamin deficiency does not affect the integrity of the erythrocyte phospholipid membrane, and therefore, acanthocytosis is not common in dogs with hypocobalaminemia.

No significant correlations were observed between folic acid concentrations and erythrocyte parameters. This probably suggests that this vitamin does not play as important a role in dogs as in humans during erythropoiesis. However, in the presence of sufficient cobalamin, the role of folic acid may be less pronounced in terms of its effect on erythropoiesis [37]. This could explain the lack of significant correlations observed in our study. In addition, dietary intake through commercially available feed and compensatory mechanisms in dogs may mitigate the effects of folic acid deficiency, resulting in less marked hematological changes.

Dogs exhibit compensatory mechanisms to mitigate the effects of folate deficiency in hematopoiesis, potentially explaining the lack of correlation. Folate is crucial for one-carbon metabolism, specifically in DNA methylation and synthesis through homocysteine remethylation to methionine. When folate is low, the body can increase choline metabolism to support methylation, partially compensating for the deficiency [38]. Folate transporters such as PCFT and RFC are also upregulated to enhance dietary folate absorption, aiding erythropoiesis [39]. Additionally, metabolic adjustments, including upregulated DNA repair proteins, help offset impaired DNA synthesis [40]. Adequate vitamin B12 further supports red blood cell production and can alleviate folate deficiency’s hematologic impact, as these vitamins are interdependent [13].

The significant correlations between cobalamin concentrations and various erythrocyte parameters underscore the importance of evaluating cobalamin status in dogs with chronic diarrhea with concurrent anemia or abnormal erythrocyte markers. According to these results, in dogs with anemia, low MCV, and/or anisocytosis in the blood smear, serum vitamin concentrations should be measured.

In cases where the owner’s financial means do not allow the measurement of these vitamins, the results of the CBC should be carefully reviewed to determine whether or not hypocobalaminemia is more likely to be present. This could aid the clinician to select more precisely a further diagnostic approach and plan the therapeutic management of the case. The presence of anisocytosis, in particular, which is significantly associated with hypocobalaminemia, may serve as a potential diagnostic indicator. The detection of anisocytosis in blood smears from dogs with chronic diarrhea could be an indication to measure cobalamin in case vitamin supplementation is needed. The relationship between normal cobalamin levels and acanthocytosis needs further investigation. While acanthocytosis may not be directly related to cobalamin sufficiency or deficiency, its presence in dogs with chronic diarrhea should prompt a thorough evaluation for concomitant liver diseases, which may contribute to the observed erythrocyte morphology [41].

The findings of the present study are consistent with the established role of cobalamin in erythropoiesis. Previous research in both human and veterinary literature has documented the hematological consequences of cobalamin deficiency, including anemia, and anisocytosis [42]. The significant correlations observed in our study support these findings and highlight the importance of cobalamin in maintaining normal hematological health.

The lack of correlation between folate concentrations and erythrocyte parameters is in contrast with the well-documented role of folate in erythropoiesis. However, similar observations have been reported in other studies where folic acid deficiency was less prevalent or had a less pronounced impact on erythropoiesis compared with cobalamin deficiency. Further research is needed to clarify the specific conditions under which folic acid deficiency may affect erythrocyte parameters in the dog.

Cobalamin plays a critical role in the conversion of homocysteine to methionine, a reaction in which folic acid is also involved. This process is crucial for DNA synthesis and cell division. In cobalamin deficiency, the resulting accumulation of homocysteine and impaired DNA synthesis lead to defective erythropoiesis, which is characterized by megaloblastic changes in erythroid precursors [43]. This mechanism in humans results in macrocytosis, anisocytosis, and reduced erythrocyte production. The latter two findings were also observed in our study.

The differences in erythropoiesis between canines and humans could explain why cobalamin deficiency, commonly associated with macrocytic anemia in humans, does not produce macrocytosis in dogs. In humans, cobalamin deficiency impairs DNA synthesis, leading to enlarged red blood cells (RBCs) due to disrupted thymidine production, which results in the release of immature RBCs [44]. In contrast, studies show that cobalamin-deficient dogs often display hematological changes like neutropenia and hypersegmented neutrophils but not macrocytosis [15]. This difference may be due to the canine bone marrow’s distinct regulatory mechanisms and compensatory responses that prevent the release of immature RBCs, along with varying interactions between nutritional deficiencies like iron or folate, which in humans can exacerbate macrocytic anemia [29,45]. Additionally, canine erythropoiesis may be less sensitive to cobalamin deficiency due to different erythropoietin regulation [46]. Thus, species-specific mechanisms in erythropoiesis likely account for these distinct hematological responses to cobalamin deficiency.

### Study Limitations

One of the main limitations of the present retrospective study is the inability to determine the cause of the enteropathy in many patients. While cobalamin and folic acid are critical for normal erythropoiesis, other factors also affect erythrocyte morphology and number and should not be overlooked. Chronic inflammation, common in dogs with chronic diarrhea, can lead to chronic anemia, characterized by orthocytic and orthochromic anemia [47]. In addition, concurrent conditions such as liver disease, kidney disease and other systemic diseases can affect erythrocyte production and morphology, potentially confounding the effects of nutrient deficiencies [48].

The study was based on serum cobalamin and folic acid concentrations as indicators of deficient or sufficient status. While these serum vitamin levels are commonly used in clinical practice, they may not always accurately reflect tissue reserves or functional deficiencies. For example, serum cobalamin levels may be influenced by recent dietary intake and other factors. Measurement of additional biomarkers, such as MMA or homocysteine, could provide a more comprehensive assessment of cobalamin status and its effect on erythropoiesis [49].

Longitudinal follow-up studies of dogs with chronic diarrhea that will include control groups would provide valuable information on the evolution of vitamin B deficiencies and their effect on erythrocyte parameters. Monitoring changes in cobalamin and folic acid levels, along with erythrocyte markers, could help to determine causal relationships and the temporal sequence of events. Furthermore, studies evaluating the effect of cobalamin and folic acid supplementation on erythrocyte parameters in dogs with chronic diarrhea would further elucidate the therapeutic possibilities of addressing these deficiencies.

Further studies investigating the biochemical pathways affected by cobalamin and folic acid deficiencies in dogs will enhance our understanding of the cellular and molecular processes involved. Investigation of the expression and activity of enzymes involved in DNA synthesis and erythropoiesis, as well as the effect of chronic inflammation on nutrient metabolism, could provide a deeper understanding of the hematological changes observed. In particular, an evaluation of the effect of the two vitamins on erythropoiesis in the dog is needed, as the results of the present study reveal a different effect of the deficiency of the two vitamins in the dog compared to humans.

## 5. Conclusions

In conclusion, this study demonstrates that cobalamin plays a key role in supporting healthy erythropoiesis in dogs and underscores the importance of regular assessment and supplementation in dogs with deficiencies. The findings also show that CBC can be a valuable tool in the diagnostic investigation of dogs with chronic diarrhea, although its use as a biomarker should be further evaluated through larger prospective studies. Although no significant link was found between folic acid levels and erythropoiesis, this suggests the interaction of nutrients is more complex than expected, requiring a thorough evaluation of all factors. Additionally, the study’s observations on erythrocyte morphology in relation to vitamin levels may assist in early diagnosis and management of cobalamin and folic acid deficiencies.

## Figures and Tables

**Table 1:** Epidemiological data of the dogs with chronic diarrhea included in the study.

Characteristic	Details
Total number of dogs	47
Gender	25 Female (53.2%) 22 Male (46.8%)
Age	Median: 5 years Range: 1–12 years
Breed Distribution	Mixed-breed: 25 dogs (53.2%) Purebred: 22 dogs (46.8%)
Purebred Breakdown	Pitbull: 6 dogs (12.8%) Labrador Retriever: 5 dogs (10.6%) Yorkshire Terrier: 3 dogs (6.4%) Other Breeds (1 dog each): Boxer, Pointer, English Setter, Golden Retriever, French Bulldog, Schnauzer, Maltese, German Shepherd (2.1% each)

**Table 2:** Correlation of cobalamin with erythrocytic parameters.

		RBC	HGB	HCT	MCV	MCH	MCHC	RDW	RETIC
B12	ρ	0.313	0.393	0.399	0.334	0.129	−0.017	−0.198	−0.174
	*p*	0.032	0.006	0.005	0.022	0.388	0.908	0.181	0.243

**Table 3:** Correlation of folate with erythrocytic parameters.

		RBC	HGB	HCT	MCV	MCH	MCHC	RDW	RETIC
Folate	rho	0.158	0.393	0.166	0.158	0.037	−0.004	−0.035	0.126
	*p*	0.288	0.006	0.265	0.290	0.806	0.977	0.815	0.398

**Table 4:** Morphological changes reported in the blood smear.

Morphological Change	Number of Cases (*n*)	Percentage of Total (%)
Anisocytosis	9	19.1%
Acanthocytosis	13	27.7%
Echinocytosis	14	29.8%
Polychromasia	7	14.9%

**Table 5:** Correlation of cobalamin with blood smear morphological changes.

Group	Total Animals	Anisocytosis	Acanthocytosis	Significant Correlation
Group A (Cobalamin < 173 pmol/L)	19	8 (42.1%)	2 (10.5%)	Anisocytosis (*p* = 0.002)
Group B (Cobalamin ≥ 173 pmol/L)	28	1 (3.6%)	11 (39.3%)	Acanthocytosis (*p* = 0.046)

## Data Availability

The data presented in this study are available on request from the corresponding author due to privacy restrictions.
